# Supplementary material for: Nanoscopic Characterization of Starch Biofilms Extracted from the Andean Tubers Ullucus tuberosus, Tropaeolum tuberosum, Oxalis tuberosa, and Solanum tuberosum
Source: Polymers (Basel). 2022 Oct 1;14(19):4116. doi: 10.3390/polym14194116 (PMC9573434; doi:10.3390/polym14194116)
Supplement: Supplementary file 1 [file polymers-14-04116-s001.zip › polymers-1917302-supplementary.pdf]

polymers

Supplementary

Nanoscopic Characterization of Starch Biofilms Extracted from the Andean Tubers *Ullucus tuberosus*, *Tropaeolum tuberosum*, *Oxalis tuberosa*, and *Solanum tuberosum*

Cynthia Pico [1,†], Jhomara De la Vega [2,†], Irvin Tubón [1], Mirari Arancibia [1,2] and Santiago Casado [1,*]

[1] Food and Biotechnology Science and Engineering Department, Technical University of Ambato, Ambato 180207, Ecuador
[2] Isabrubotanik S.A., Ambato 180150, Ecuador
* Correspondence: s.casado@uta.edu.ec; Tel.: +593-2400987 (ext. 5509)
† These authors contributed equally to this work.

Citation: Pico, C.; De la Vega, J.; Tubón, I.; Arancibia, M.; Casado, S. Nanoscopic Characterization of Starch Biofilms Extracted from the Andean Tubers *Ullucus tuberosus*, *Tropaeolum tuberosum*, *Oxalis tuberosa*, and *Solanum tuberosum*. *Polymers* **2022**, *14*, 4116. https://doi.org/10.3390/polym14194116

Academic Editor: Vincent Chan

Received: 30 August 2022
Accepted: 26 September 2022
Published: 1 October 2022

Publisher's Note: MDPI stays neutral with regard to jurisdictional claims in published maps and institutional affiliations.

Copyright: © 2022 by the authors. Submitted for possible open access publication under the terms and conditions of the Creative Commons Attribution (CC BY) license (https://creativecommons.org/licenses/by/4.0/).

Supplementary Materials:

S.3.1. Macroscopic optical differences between films containing mouthwash

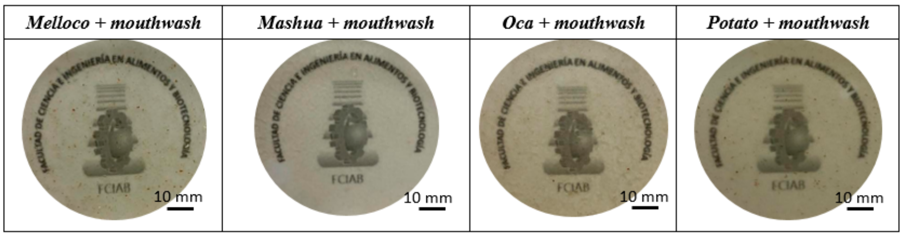

Figure S1. Visual appearance and apparent transparency of starch films with mouthwash extracted from each of the Andean tubers analyzed.

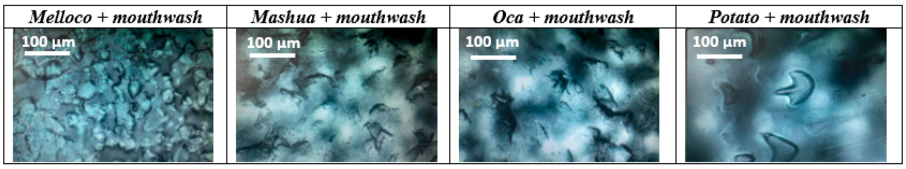

Figure S2. Micrographs of starch films extracted from each of the Andean tubers analyzed, after addition of a mouthwash to each one, in reflection using white light transmission illumination.

S.3.2. Nanoscopic characterization

S.3.2.1. Topography

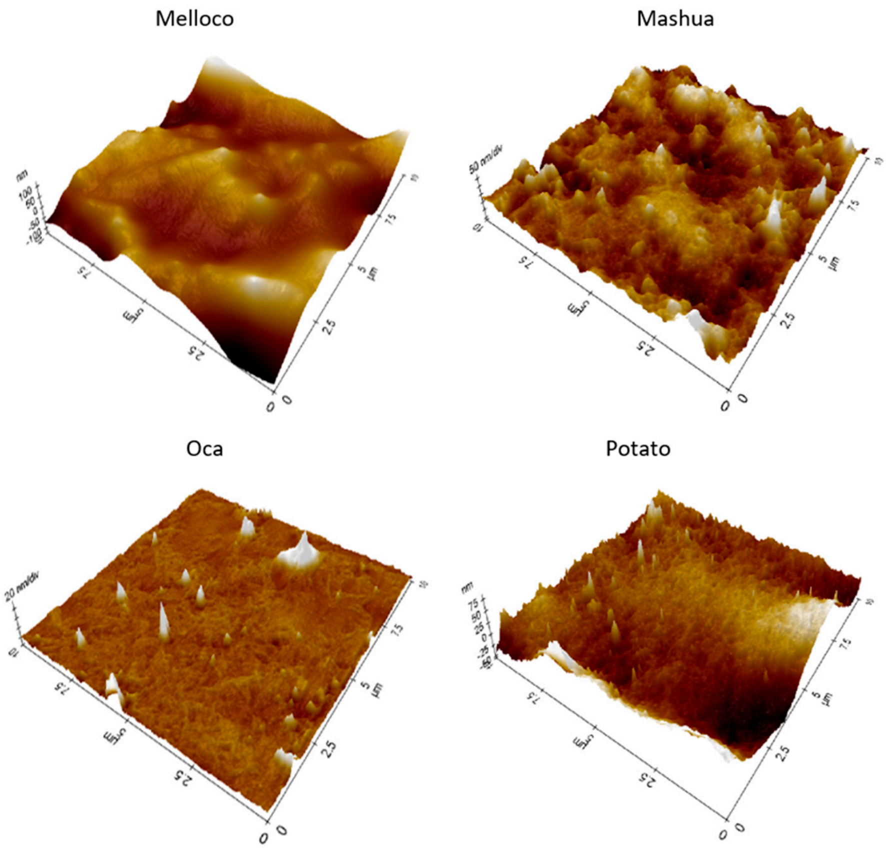

Figure S3. Atomic Force Microscopy topography 3D images of 10 x 10 μm² range.

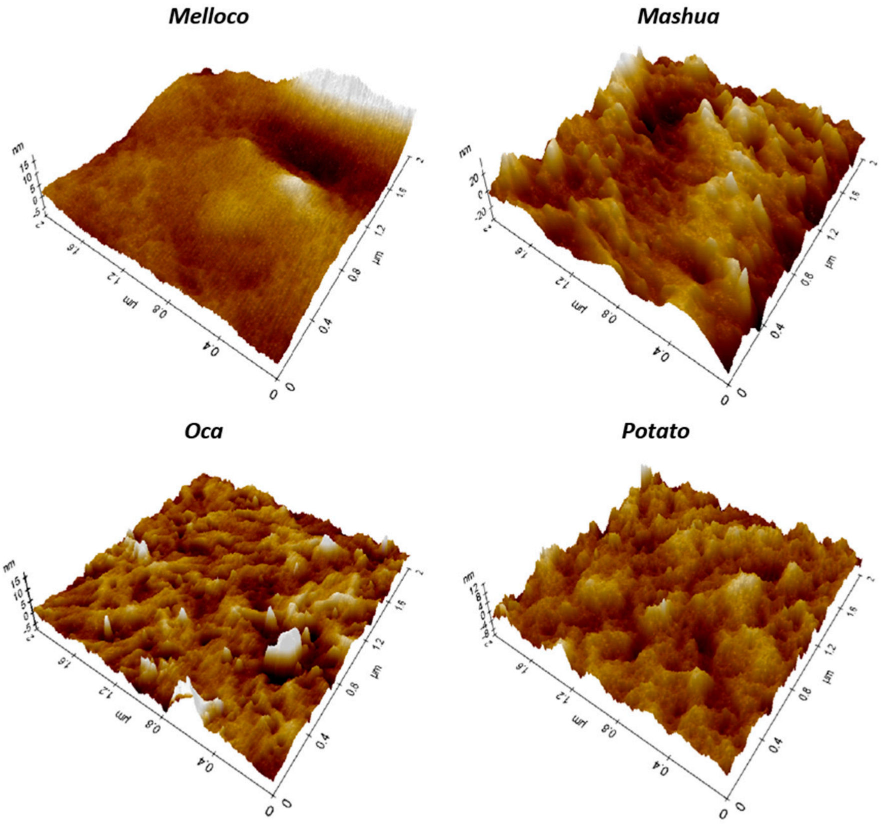

Figure S4. Atomic Force Microscopy topography 3D images of 2 x 2 μm² range.

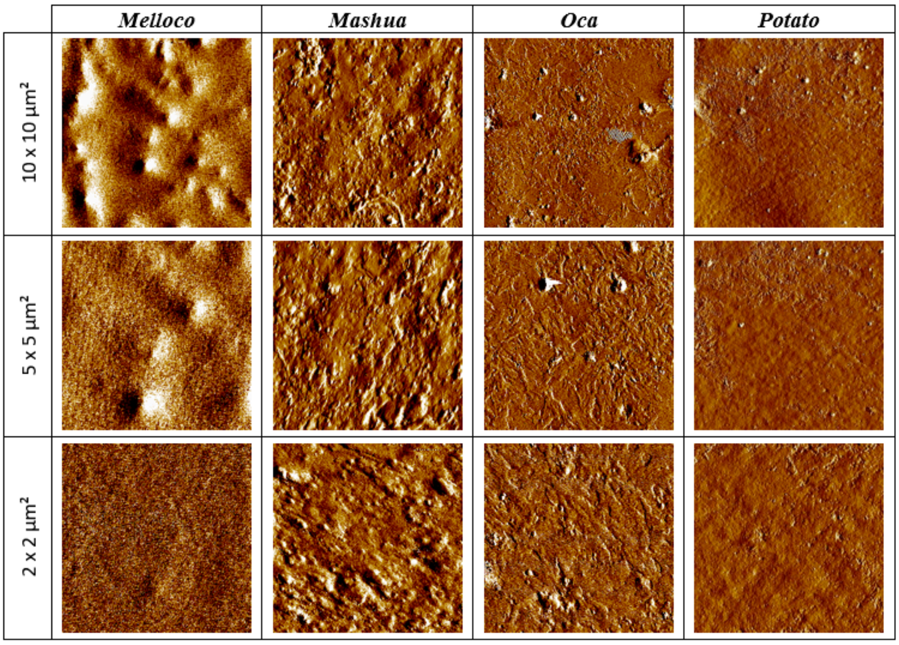

Figure S5. Atomic Force Microscopy amplitude (lock-in, error signal) measured in tapping mode on the surface of biofilms extracted from the Andean tubers melloco, mashua, oca, and potato, at different scan areas.
